# In Vitro Alpha-Amylase and Alpha-Glucosidase Inhibitory Activity and In Vivo Antidiabetic Activity of *Withania frutescens* L. Foliar Extract

**DOI:** 10.3390/molecules26020293

**Published:** 2021-01-08

**Authors:** Hamza Mechchate, Imane Es-safi, Abdelhadi Louba, Ali S. Alqahtani, Fahd A. Nasr, Omar M. Noman, Muhammad Farooq, Mohammed S. Alharbi, Abdulaziz Alqahtani, Amina Bari, Hicham Bekkari, Dalila Bousta

**Affiliations:** 1 Laboratory of Biotechnology, Environment, Agrifood, and Health, University of Sidi Mohamed Ben Abdellah, FSDM-Fez, Fez 30003, Morocco; Imane.essafi1@usmba.ac.ma (I.E.-s.); abdelhadi.louba@gmail.com (A.L.); aminabari3@gmail.com (A.B.); Hicham.bekkari@usmba.ac.ma (H.B.); dalila.bousta@usmba.ac.ma (D.B.); 2 Medicinal Aromatic, and Poisonous Plants Research Centre, College of Pharmacy, King Saud University, Riyadh 11451, Saudi Arabia; alalqahtani@ksu.edu.sa (A.S.A.); fnasr@ksu.edu.sa (F.A.N.); Onoman@ksu.edu.sa (O.M.N.); mohsalharbi@ksu.edu.sa (M.S.A.); 3 Department of Pharmacognosy, College of Pharmacy, King Saud University, Riyadh 11451, Saudi Arabia; 439106051@ksu.edu.sa; 4 Department of Zoology, College of Science, King Saud University, P.O. Box 2455, Riyadh 11451, Saudi Arabia; fmuhammad@ksu.edu.sa

**Keywords:** *Withania frutescens* L., diabetes mellitus, alloxan monohydrate, glibenclamide, antidiabetic, antihyperglycemic, alpha-amylase, alpha-glucosidase

## Abstract

*Withania frutescens* L. is a wild perennial woody plant used by the local population for diverse therapeutic purposes. This work aims to study for the first time the potential inhibitory effect of this plant hydroethanolic extract on α-amylase and α-glucosidase activities using in vitro methods and its antidiabetic and antihyperglycemic activities using alloxan-induced diabetic mice as a model for experimental diabetes. Two doses were selected for the in vivo study (200 and 400 mg/kg) and glibenclamide, a well-known antidiabetic drug (positive control) in a subacute study (28 days) where the antihyperglycemic activity was also assessed over a period of 12 h on diabetic mice. The continuous treatment of diabetic mice with the extract of *Withania frutescens* for 4 weeks succeeded to slowly manage their high fasting blood glucose levels (after two weeks), while the antihyperglycemic test result revealed that the extract of this plant did not control hyperglycemia in the short term. No toxicity signs or death were noted for the groups treated with the plant extract, and it shows a protective effect on the liver and kidney. The in vitro assays demonstrated that the inhibition of alpha-amylase and alpha-glucosidase might be one of the mechanisms of action exhibited by the extract of this plant to control and prevent postprandial hyperglycemia. This work indicates that W. frutescens have an important long term antidiabetic effect that can be well established to treat diabetes.

## 1. Introduction

Diabetes mellitus is a chronic condition that affects normal metabolism of carbohydrates, fats, and proteins, triggering numerous changes in the body’s biochemical system that contribute to hyperglycemia, which is the disease’s characteristic [1]. The prevalence of diabetes worldwide raised from 2.8% in 2000 to 4.4% in 2030. It is estimated that the overall number of diabetic people will jump to over 366 million cases in 2030 [2]. Diabetes comes with several complications that threatened people’s lives like chest pain, heart attack, stroke, cardiovascular problems (coronary artery disease), atherosclerosis, and neuropathy [3]. Multiple synthetic oral hypoglycemic agents are used to overcome elevated blood glucose levels, such as sulfonylureas, biguanides, and inhibitors of alpha-glucosidase. Still, their extended use has revealed more of their several side effects [4] like hypoglycemia, headache, dizziness, nausea, and weight gain [5]. Those side effects made it a priority obligation to find new effective and safer alternatives [6]. The attention of researchers has now turned to medicinal plants because they are known to be effective, available, and usually have fewer side effects [7]. In many developed and underdeveloped countries, plants have been used from time immemorial as an alternative form of medication for different physiopathological diseases, including diabetes mellitus [8]. 

The plant kingdom’s vast diversity encompasses enormous plants with various medicinal proprieties, among them is *Withania frutescens* L. (Family: Solanaceae, genera: *Withania)* known to contain several bioactive compounds in its composition (phenolic acids, flavonoids, tannins, terpenoids, saponins and polyphenols), responsible for multiple activities like antioxidant, antifungal, and antimicrobial activity detailed in our previous research [9]. The same genera also encompasses other plants known for their anti-inflammatory, anti-tuberculosis, immunomodulatory [10], and even antidiabetic activities [11].

In this study, we are investigating for the first time the antidiabetic and antihyperglycemic activity of *W. frutescens* leaves on alloxan-induced diabetic mice for 4 weeks along with a toxicity assessment on the effect of the treatments on the overall health of the mice and we are testing the plant possible inhibitory effect against the alpha-glucosidase and alpha-amylase enzymes

## 2. Results and Discussion

### 2.1. Phytochemical Composition and the Acute and Sub-Acute Toxicity Study

The phytochemical composition and the acute and subacute toxicity of *W. frutescens* hydroethanolic extract were already being studied in detail in our previous research [12]. 

Acute oral toxicity of the extract was evaluated in mice according to the Organization of Economic Cooperation and Development (OECD) guidelines 423 [13] with a single oral administration of the extracts at the doses of 400, 1000, and 2000 mg/kg (and the animals were monitored for 14 days). The subacute toxicity study followed the OECD guidelines 407 [13] with a daily administration of *Withania frutescens* leaves extract (WFLE) for 28 days at the dose of 400 and 2000 mg/kg/day. Both studies confirmed the plant extract’s total safety at the studied doses under both acute and subacute toxicity conditions.

The phytochemical analysis was performed and revealed different interesting molecules like (a) terpinene-4-ol, a compound found in multiple plants and has antimicrobial effects against various bacteria and microorganisms [14], (b) ferrocene, which possesses an anti-proliferative effect against lymphocytic leukemia [15] and also approved antidiabetic effects [16], (c) phenazine, a molecule with antimicrobial proprieties [17] and also affects the viability of pancreatic β-cells and has an impact on the secretion of insulin [18]. These compounds may be behind the antidiabetic effect of the extract and needs further investigation to confirm it.

### 2.2. Effect of WFLE on FBG Level

**Figure 1:**
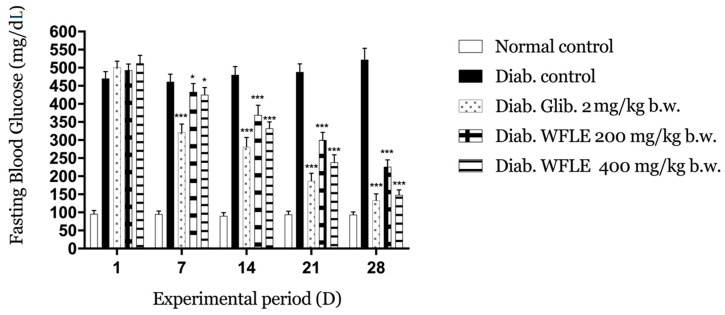
Effect of the continuous administration of *Withania frutescens* leaves extract (WFLE) on fasting blood glucose (FBG) on alloxan-induced diabetic mice for four weeks (n = 5 mice). * *p* < 0.05, *** *p* < 0.001 compared to diabetic control.

Based on previous pharmacological tests and a toxicity study, two doses (200 and 400 mg/kg) were chosen as the lowest most effective doses to treat the mice with induced diabetes [12,19]. Figure 1 presents the treatment’s effect on fasting blood glucose (FBG) during the study period of 4 weeks of daily oral administration. In this type of experimental diabetes (chemically induced), we used alloxan monohydrate, a glucose toxic analog that uses the same glucose transporter 2 (GLUT2) to penetrate into pancreatic β-cells and drive them to apoptosis following its free radical formation ability. Degeneration of the β-cells responsible for the production of insulin results in an elevated fasting blood glucose [20]. In our study and following the intraperitoneal injection of alloxan monohydrate at the dose of 180 mg/kg, an increased blood glucose level (+450 mg/dL) was noted.

The FBG of diabetic mice was estimated on day 1, 7, 14, 21, and 28 of the treatment. Both WFLE doses show a statistically significant reduction with diabetic control mice (*p* < 0.05) within the first week but not compared to the powerful reduction noted with the glibenclamide treatment (*p* < 0.001). There was also no significant difference between the decrease in the FBG of the two doses during this week (433 ± 23 mg/dL for the dose of 200 mg/kg and 425 ± 20 mg/dL for the dose of 400 mg/kg against 321 ± 23 mg/dL noted for glibenclamide). However, this reduction becomes very significant during the next three weeks for both doses (*p* < 0.001) and the difference in the decrease in the FBG level becomes evident by the end of the test with 266 ± 19 mg/dL noted for the dose of 200 mg/kg and 148 ± 14 for the dose of 400 mg/kg against the best FBG level marked with glibenclamide 133 ± 18 mg/dL. Those results show that WFLE extracts exhibit better fasting blood glucose level activity with the dose of 400 mg/dL, a reduction that may reach the glibenclamide effect with a longer time of use.

**Figure 2:**
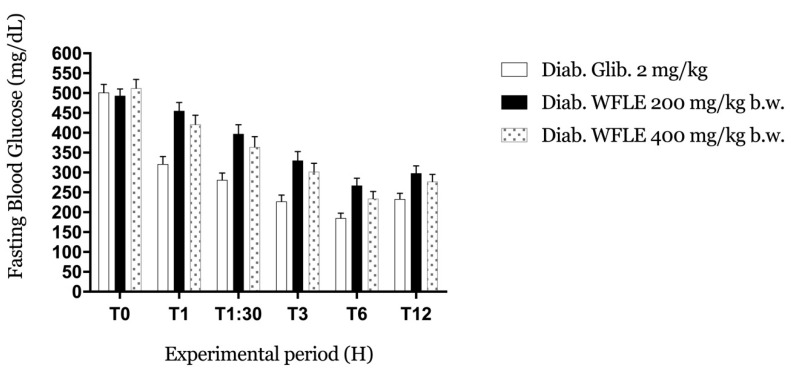
Antihyperglycemic effect of WFLE on alloxan-induced diabetic mice during 12 h (n = 5 mice).

As for the antihyperglycemic results detailed in Figure 2, during the 12 h period, the different doses did not show any remarkable effect in the management of BGL in a short time. Compared to glibenclamide, the two doses exhibited a very moderate effect. The decrease in FBG stopped between the 6th and 12th hour and started to rise again, indicating the end of the extract’s effect.

Those results indicate that the antidiabetic effect of *W. frutescens* needs a longer time to appear and take place, which suggests that the different molecules contained in the extract act indirectly against the mains actors responsible for high blood glucose levels. 

Table 1 displays the bodyweight development and biochemical parameters of alloxan-induced diabetic mice during the experimental period of 4 weeks. Untreated diabetic mice have reported a substantial drop in body weight concomitant with hyperglycemia. The decline in body weight of diabetic mice is attributed to the excessive catabolism of fats and structural proteins used as an energy source because of the lack of availability of carbohydrates. Insulin plays a vital role in regulating protein synthesis and proteolysis in the skeletal muscle, and the observed effect is mainly due to its deficiency [21,22]. Treatment of diabetic mice with WFLE and glibenclamide for four weeks improved their body weight concomitant with the stabilization of the hyperglycemic state. In Table 2, the biochemical parameters indicate an elevated serum ASAT and ALAT levels in the untreated diabetic mice, which is a marker of cellular leakage and loss of hepatic cell membrane functional integrity, which implies hepatocellular injury [23]. After the treatment of diabetic mice with different doses of WFLE, a significant decrease in ASAT and ALAT levels compared to those of the diabetic control groups was observed. The results may indicate a potential hepatoprotective activity of the extract maybe due to its antioxidant, anti-inflammatory and healing proprieties [9,19]. High levels of urea and creatinine in the blood were also observed in the diabetic untreated groups indicating a renal injury [24], and by administering WFLE to diabetic mice, their levels were normally maintained.

### 2.3. In Vitro Assays

Plants have a long history of use as a treatment for diabetes, and recent studies confirmed their healing potential and also the mechanism by which they exhibit their activities. Plants metabolites can act through different pathways such as increasing insulin secretion and glucose uptake by various cells and inhibiting the glucose production and absorption, and they can act indirectly by preventing and treating oxidation and inflammation as they were recently related to the development and aggravation of the overall diabetic state [25].

One of the important approaches adopted as treatment of diabetes is the inhibition of glucose absorption. By inhibiting the digestive enzymes responsible for the hydrolysis of polysaccharides to small absorbable fragments, we prevent postprandial high blood glucose. An example of this group of inhibitors *α*-glucosidase and *α*-amylase inhibitors. WFLE potential inhibitory effect will be tested against those two enzymes to possibly reveal one or more modes of action of this plant.

#### 2.3.1. Alpha-Amylase Inhibitory Effect

Due to its major role in the breakdown of polysaccharides, alpha-amylase is considered one of the most important enzymes in the digestion process found primarily in the saliva and pancreatic juice [26]. Targeting this enzyme and inhibiting it is one of the possible solutions to prevent high postprandial blood glucose [27].

**Figure 3:**
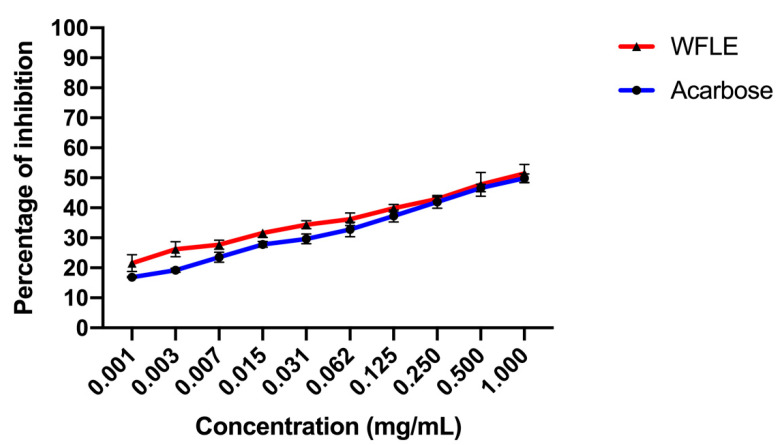
Alpha-amylase inhibitory effect results (n = 3).

Alpha-amylase inhibition potential of WFLE is demonstrated in Figure 3. The inhibition of the enzyme appears to be dose-related as the concentration of the extract clearly affects the amount of enzyme inhibited. The calculated IC50 showed that WFLE inhibition potential is better than acarbose, with an IC50 of 0.40 ± 0.124 mg/mL noted for WFLE against 0.717 ± 0.054 mg/mL noted for acarbose.

Those results propose that one potential mode of action exhibited by WFLE is the inhibition of the Alpha-amylase enzyme and limiting glucose absorption.

#### 2.3.2. Alpha-Glucosidase Inhibitory Effect

The *α*-glucosidase enzyme is also one of the essential enzymes of the digestion, located in the mucosal brush border of the small intestine. Its role consists of the processing and degradation of complex carbohydrates into small, simple and absorbable ones. Its inhibition is an effective solution to delay glucose absorption and also prevent high postprandial blood glucose levels, which may probably suppress diabetes progression.

**Figure 4:**
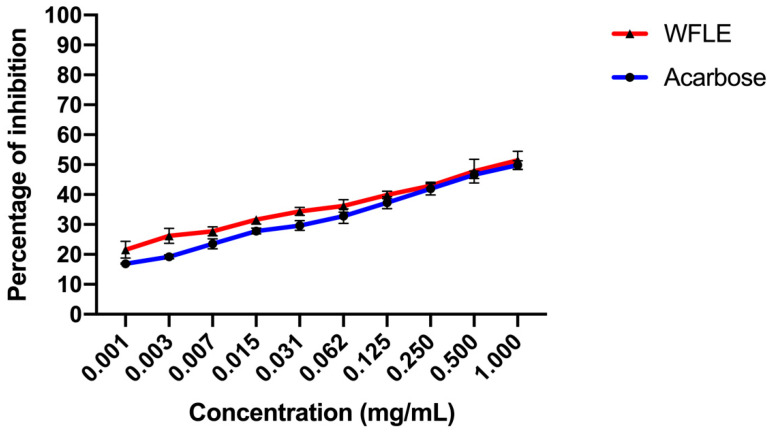
Alpha-glucosidase inhibitory effect results (n = 3).

Figure 4 illustrates the alpha-glucosidase inhibition activity of WFLE. The inhibition effect is correlated to the concentration of the WFLE as the highest concentrations exhibited the highest inhibition activity. The calculated IC50 showed that the inhibition potential of acarbose (IC50 0.084 ± 0.017 mg/mL) is better than that of WFLE (IC50 0.180 ± 0.018 mg/mL). Despite its moderate activity, the inhibition of alpha-glucosidase remains one of the proposed potential mode of action exhibited by WFLE.

## 3. Materials and Methods

### 3.1. Materials and Reagents

Alpha-glucosidase (CAS: 9001-42-7), alpha-amylase (CAS: 9000-85-5), alloxan monohydrate (CAS: 2244-11-3) and glucose (CAS: 50-99-7) were obtained from Sigma-Aldrich (St. Louis, MO, USA). Glibenclamide was purchased from a local pharmacy and all other chemicals and reagents used in this study were analytical grade.

### 3.2. Plant

*Withania frutescens* L. leaves were collected from Fez-Meknes region, Morocco (March 2019). The plant authentication and identification were conducted by professor Amina Bari (botanist, FSDM, Fez). A representative sample (voucher specimens) was prepared and stored in the herbarium of the LBEAS laboratory under the number BPRN69.

### 3.3. Preparation of the Crude Extract

The leaves were gently rinsed and dried at room temperature. An electric grinder was used for crushing the dried leaves to a fine powder.

The extraction begins with hydro-ethanolic maceration: 100 mL of 70/30 (Ethanol: distillated water) hydroethanolic solution with 10 g of plant powder for 24 h at room temperature. Afterward, the mixture was filtered through Whatman filter paper and concentrated using a rotary evaporator (Final yield 13.6%). The dry crude extract (Solid) was stored at 4 °C until future use.

### 3.4. Animals Used

Swiss albino mice were used in the study. They were obtained from the animal facility of the LBEAS Laboratory, weighing between 22 and 27 g and aged eight weeks. Mice were housed in cages (five mice/cage) in controlled conditions with a temperature of 23 ± 2 °C and a light–dark cycle of 12 h. Before starting the test, the acclimatization period for the animals was two weeks. During the test period, the animals had free access to food and water.

The methods used in this study are in accordance with international directives for the use of laboratory animals and the institutional ethical committee recommendations (N-ANI-BPRN-135).

### 3.5. Antidiabetic and Antihyperglycemic Activity

**Figure 5:**
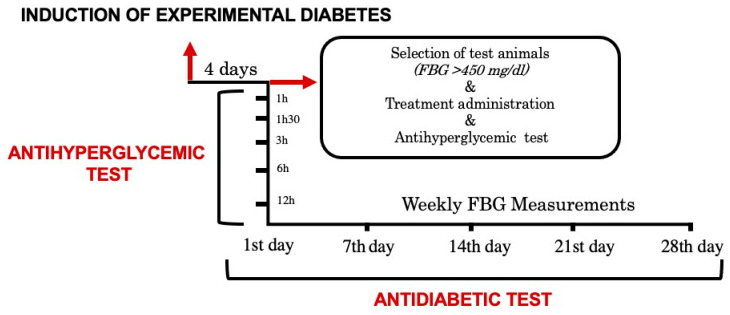
Experimental design.

The antidiabetic effect of WFLE was evaluated for 28 days (subacute study) on alloxan-induced diabetic mice. Along with this test, the antihyperglycemic activity was also assessed. During the study period, Bodyweight measurements were performed weekly, and the biochemical parameters changes were measured at the end of the study. The experimental design is demonstrated in Figure 5.

#### 3.5.1. Induction of Experimental Diabetes

Experimental diabetes was induced by an intraperitoneal injection of freshly prepared alloxan monohydrate [20] at the dose of 180 mg/kg to at least 12 h-fasted animals. Following this injection, a large proportion of the beta cells would be subjected to apoptosis (alloxan monohydrate mode of action), and this would cause the liberation of its content in the blood, especially insulin. The high insulin level in the blood would cause a hypoglycemic shock to the animals and may lead to death. To avoid this shock, a glucose preparation (0.2 mL / 4 g/L concentration) was orally administrated to the animals.

Four days after the induction and after stabilization of blood glucose levels, measurements were taken, and animals with FBG (fasting blood glucose) above 450 mg/dL were selected.

#### 3.5.2. The Experimental Model of Fasting Glucose Measurement

The sub-acute study involved the repeated administration of the treatments for 28 days, and the blood glucose levels were estimated on days 7, 14, 21, and 28.

Mice selected for the study were divided into five different groups of five mice each.

Group I: normal control treated with distilled water (D.W.) (0.2 mL/day).
Group II: diabetic control treated with D.W. (0.2 mL/day).
Group III: diabetic mice treated with glibenclamide (2 mg/kg/day b.w.).
Group IV: diabetic mice treated with WFLE (200 mg/kg/day b.w.).
Group V: diabetic mice treated with WFLE (400 mg/kg/day b.w.).

The data were represented as mean blood glucose levels. Bodyweights of the mice were also noted during this study period. On the last day of the study, mice were sacrificed by cervical decapitation following anesthesia to collect blood to estimate biochemical parameters.

#### 3.5.3. Evaluation of the Antihyperglycemic Activity

The antihyperglycemic activity was assessed on the 1st day of the antidiabetic study. After the first measurement on the 1st day and selection of the groups, the treatments were administered right away, and series of measures were conducted after 1 h, 1 h 30, 3 h, and 12 h to evaluate the response of the high blood glucose animal to the different treatments in a short and long time.

All treatments were given orally to animals by intragastric gavage using a stainless-steel bulb tipped gavage needle attached to a syringe to deliver the compound into the stomach.

Prior to the administration, the solution was prepared by dissolving the crude extract in distilled water. The prepared solution was kept in a refrigerator after each oral administration.

### 3.6. In-Vitro Assays

#### 3.6.1. α-Amylase Inhibitory Assay

The test was realized pursuing Mitra et al.’s protocol [28]. To prepare the substrate solution, a solution of 0.5 M Tris–HCl buffer at pH 6.9 was mixed with 0.01 M CaCl2 (0.2 mL) and 2 mg of starch. The substrate solution was distributed into test tubes, boiled (for 5 min) and preincubated (at 37 °C for 5 min). Using DMSO, WFLE extract was dissolved and prepared at different concentrations of 1, 3, 7, 15, 31, 62, 125, 250, 500 and 1000 μg/mL. The plant extract solution (0.2 mL) at different concentrations was added to the test tube containing the substrate solution, and later on, the porcine pancreatic amylase (0,1 mL in Tris–HCl buffer (2 units/ mL)) was added. This reaction was performed out at 37 °C for 10 min and then stopped by adding 0.5 mL of 50% acetic acid in each test tube. A centrifugation was carried on (3000 rpm for 5 min at 4 °C) and the supernatant optic density was at 595 nm using a spectrophotometer. For this assay, acarbose was used as a positive control (α-amylase inhibitor). The experiments were repeated three times for each concentration.

To calculate the α-amylase inhibitory activity, this formula was used:

Extract inhibitory activity = [(X_A_ − X_B_)/X_A_] × 100.

X_A_ is the absorbance of the control (100% enzyme activity) and X_B_ is the absorbance of the sample.

After determining the α-amylase inhibitory activity of the different concentrations, the IC50 values were determined for the acarbose and WFLE extract (concentration required to inhibit 50% of α-amylase).

#### 3.6.2. α-Glucosidase Inhibitory Assay

The test was realized pursuing the protocol of Pistia-Brueggeman and Hollingsworth [29]. An amount of 50 μL of WFLE extract was prepared at various concentrations (1, 3, 7, 15, 31, 62, 125, 250, 500 and 1000 μg/mL) and incubated with the solution containing 10 μL of α-glucosidase (maltase) 1 U/mL and 125 μL of 0.1 M phosphate buffer (pH 6.8) for 20 min at 37 °C. To start the reaction, a solution of 20 μL of 1 M pNPG (4-Nitrophenyl-β-d- glucopyranoside) (substrate) was added then incubated for half-hour. To terminate the reaction, 50 μL of 0.1 N Na2CO3 was added. The optical density was measured at 405 nm using a spectrophotometer. For this assay, acarbose was used as a positive control (α-amylase inhibitor). The experiments were repeated three times for each concentration.

The α- Glucosidase inhibitory activity was calculated by using the following formula:

Extract inhibitory activity = [(X_A_ − X_B_)/X_A_] × 100.

X_A_ is the absorbance of the control (100% enzyme activity) and X_B_ is the absorbance of the sample.

After determining the α-glucosidase inhibitory activity of the different concentrations, the IC50 values were determined for the acarbose and WFLE extracts (concentration required to inhibit 50% of α-glucosidase).

### 3.7. Statistical Analysis

The statistical analysis was done with Graph Pad Prism version 8.0 for Windows. Values are expressed as the mean±SD, and the difference between groups was assessed by one-way analysis of variance (ANOVA).

The IC50 is calculated using linear regression by plotting x-y and fitting the data with a straight line. IC50 value is then estimated using the fitted line, i.e.,

Y = a × X + b; IC50 = (0.5 − b)/a

## 4. Conclusions

The results of this study confirmed the traditional use of this plant for the management of diabetes. However, it is observed that *W. frutescens* antidiabetic activity starts to be noticeable after the second week of the study, suggesting that the effect needs time to take place. The dose of 400 mg/kg exhibited the best effect with a perfect toxicity profile. One of the proposed mechanisms of action exhibited by this plant is the inhibition of two digestive enzymes responsible for the breakdown and the absorption of carbohydrates, demonstrating a better control of postprandial glycemia.

## Figures and Tables

**Table 1:** Effect of WFLE on bodyweight development.

Treatments	Bodyweight Development				
	1st Day	1st Week	2nd Week	3rd Week	4th Week
Normal control	23.4 ± 2.3	24.7 ± 2.5 *	25.2 ± 2.4 ***	26.9 ± 2.3 ***	27.2 ± 2.5 ***
Diab. Control	23.8 ± 2.1	21.7 ± 3.2	20.1 ± 2.7	19.2 ± 2.4	17.7 ± 2.2
Diab. Glib. 2 mg	24.3 ± 2.6	23.1 ± 1.6	24.2 ± 2.3 **	25.8 ± 2.5 ***	25.9 ± 1.77 ***
Diab. WFLE 200 mg/kg	24.8 ± 1.9	24.1 ± 2.0	24.9 ± 1.8 *	25.8 ± 2.1 ***	26.7 ± 1.8 ***
Diab. WFLE 400 mg/kg	23.9 ± 2.1	23.3 ± 2.2	24.8 ± 1.9 *	25.6 ± 2.1 ***	27.3 ± 2.4 ***

(n = 5 mice). * *p* < 0.05, ** *p* < 0.01, *** *p* < 0.001 compared to diabetic control. Diab: diabetic group Glib: glibenclamide.

**Table 2:** Effect of WFLE on biochemical parameters.

Treatments	Biochemical Parameters			
	ASAT	ALAT	UREA	CREATININE
Normal control	253 ± 23.73	45 ± 7.58	0.24 ± 0.04	3.2 ± 0.44
Diab. Control	502 ± 38.85	134 ± 11.26	0.63 ± 0.06	5.8 ± 0.83
Diab. Glib. 2 mg	298 ± 24.66 ***	77 ± 8.8 ***	0.28 ± 0.04 ***	4.2 ± 0.44 **
Diab. WFLE 200 mg/kg	211 ± 23.6 ***	47 ± 8.2 ***	0.25 ± 0.04 ***	3.9 ± 0.36 ***
Diab. WFLE 400 mg/kg	198 ± 22.1 ***	50 ± 6.7 ***	0.29 ± 0.05 ***	3.6 ± 0.28 ***

(n = 5 mice). ** *p* < 0.01, *** *p* < 0.001 compared to diabetic control. ASAT: aspartate transaminase; ALAT: alanine transaminase; Diab: diabetic group Glib: glibenclamide.

## Data Availability

The data that support the findings of this study are available from the corresponding author, upon reasonable request.

## Abbreviations

WFLE	*Withania frutescens* leaves extract
FBG	fasting blood glucose
OECD	Organization of Economic Cooperation and Development
D.W.	distilled water
ANOVA	analysis of variance
